# Hook2, a microtubule-binding protein, interacts with Par6α and controls centrosome orientation during polarized cell migration

**DOI:** 10.1038/srep33259

**Published:** 2016-09-14

**Authors:** Emilie Pallesi-Pocachard, Elsa Bazellieres, Annelise Viallat-Lieutaud, Marie-Hélène Delgrossi, Magali Barthelemy-Requin, André Le Bivic, Dominique Massey-Harroche

**Affiliations:** 1 Aix-Marseille Univ, CNRS, UMR 7288, Developmental Biology Institute of Marseille (IBDM), case 907, 13288 Marseille, cedex 09, France

## Abstract

Polarity protein complexes function during polarized cell migration and a subset of these proteins localizes to the reoriented centrosome during this process. Despite these observations, the mechanisms behind the recruitment of these polarity complexes such as the aPKC/PAR6α complex to the centrosome are not well understood. Here we identify Hook2 as an interactor for the aPKC/PAR6α complex that functions to localize this complex at the centrosome. We first demonstrate that Hook2 is essential for the polarized Golgi re-orientation towards the migration front. Depletion of Hook2 results in a decrease of PAR6α at the centrosome during cell migration, while overexpression of Hook2 in cells induced the formation of aggresomes with the recruitment of PAR6α, aPKC and PAR3. In addition, we demonstrate that the interaction between the C-terminal domain of Hook2 and the aPKC-binding domain of PAR6α localizes PAR6α to the centrosome during cell migration. Our data suggests that Hook2, a microtubule binding protein, plays an important role in the regulation of PAR6α recruitment to the centrosome to bridge microtubules and the aPKC/PAR complex. This data reveals how some of the polarity protein complexes are recruited to the centrosome and might regulate pericentriolar and microtubule organization and potentially impact on polarized migration.

The apical polarity PAR3/PAR6/aPKC complex regulates many cellular processes in addition to apical-basal cell polarity, including polarized cell migration, axonal outgrowth and even ciliogenesis (for review see^1^). Both PAR6α and aPKC are necessary for the correct orientation of the centrosome and the Golgi apparatus towards the migration front during polarized cell migration. However, the mechanism through which the PAR6/aPKC complex communicates with the microtubule network and regulates Golgi/MTOC (microtubule-organizing center) orientation is an important unresolved issue^2^. In migrating astrocytes, the activation of Cdc42 at the leading edge results in the recruitment of PAR6/aPKC, which in turn can act upon the microtubule network through dynein to reorient the Golgi/MTOC complex^3^. The PAR6/aPKC complex also regulates the localization of Disc Large 1 and APC (adenomatosis polyposis coli), both of which are linked to microtubule plus ends^4^. In addition to the localization of the PAR complex at the leading edge, recent studies have found that aPKC, PAR6α and PAR3 are associated with the centrosome and peri-centriolar material^5^,^6^,^7^ suggesting that these proteins may play a role in centrosome organization.

Hook2 is a member of the Hook protein family, which generally functions by connecting microtubules to sub-cellular structures^8^,^9^. Hook proteins participate in numerous cellular processes including endosome/lysosome processing^10^,^11^,^12^,^13^, the organization and maintenance of Golgi apparatus and centrosomal function^9^,^14^. Recently, we demonstrated that Hook2 is also involved in the first steps of ciliogenesis in association with PCM1 and Rab8a^15^. Here, using a combination of approaches we show that Hook2 binds to the aPKC-binding domain of PAR6α for centrosomal recruitment. In addition, depletion of Hook2 randomized centrosome orientation during polarized migration. Our data indicate that Hook2 is a critical protein to link the PAR polarity complex and MTOC/centrosome allowing the transmission of peripheral signals from the polarity complexes to the cell MTOC during the appropriate Golgi re-orientation that occurs during directional migration.

## Results and Discussion

### Hook2 controls Golgi orientation during polarized cell migration

Hook2 accumulates at the centrosome^16^ and its depletion in ARPE-19 cells leads to loss of peri-centriolar material and blocks ciliogenesis^15^. Thus, we postulated that Hook2 might play additional roles in other centrosome processes such as polarized migration in which cells reorient both their Golgi complex and their centrosome towards the front edge in a dynein-mediated process^3^. To test this hypothesis we used a wound-healing assay on MCF7 cells and a transient depletion of Hook2 by a small interfering RNA (siRNA). This strategy strongly reduced Hook2 expression to 20% of the control after 3 days (supplementary Fig. S1a) without affecting the cellular levels of the two other mammalian Hook family proteins, Hook1 and 3. These results indicate that any phenotype further observed was not due to effects on the other Hook paralogues (see supplementary Fig. S1b and S1c)^9^. In addition we used two independent siRNAs targeting Hook2 and observed the same loss of Hook2 protein (Fig. S1a). In subsequent experiments we used these two siRNAs (H1, H2) either independently or in combination for Hook2 depletion (indicated as siHook2). Hook2 rescue was not possible as its exogenous expression in a wild type background induced aggresomes^17^.

**Figure 1:**
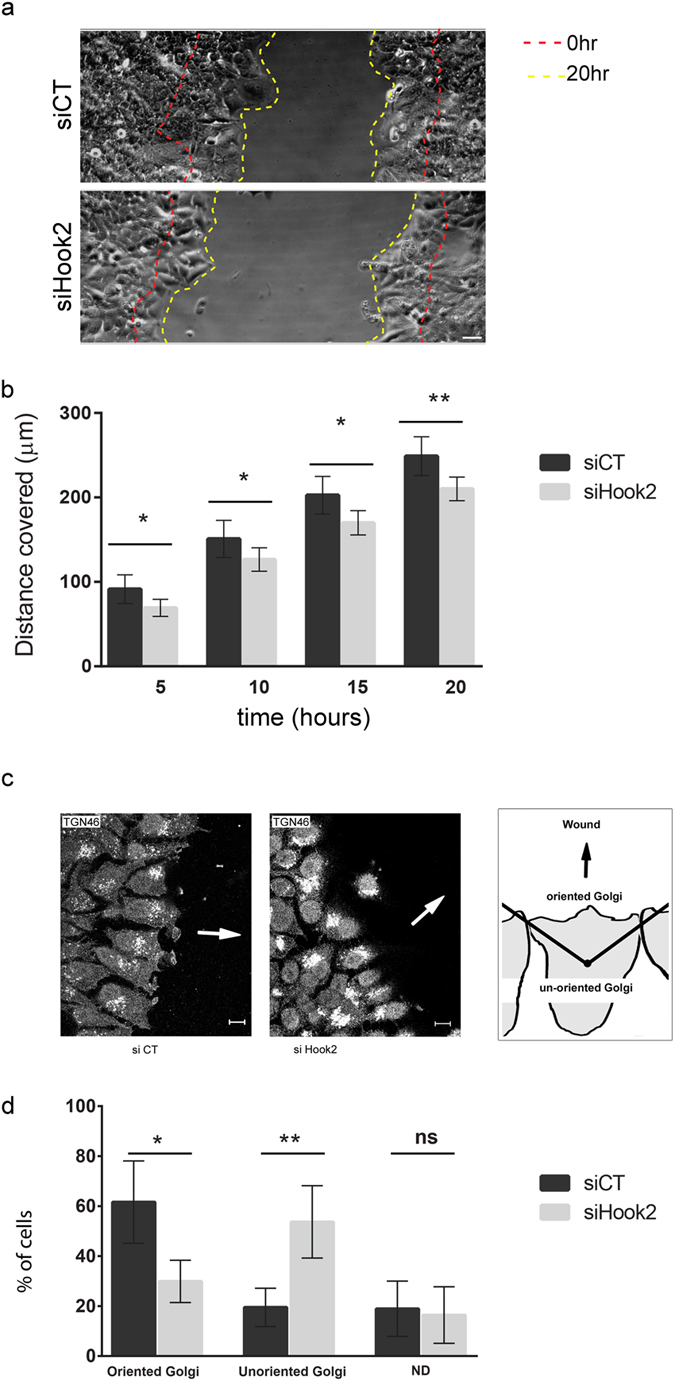
Hook2 controls Golgi orientation during polarized cell migration. (**a**) MCF7 cells were transiently transfected with siCT and siHook2 (H1 + H2) and motility of the transfected cells was illustrated by phase contrast images just after a wound healing assay (0 hour) and after 20 hours on cell monolayer (Bar 40 μm). (**b**) The estimation of cell-migration capacity was obtained by measurement of the distance covered 5, 10, 15 and 20 hours after scratching. (*n* = 3; *p = 0.0206 for 5 hours; *p = 0.0430 for 10 hours; *p = 0.0129 for 15 hours; **p = 0.0055 for 20 hours). (**c**) Golgi reorientation was analyzed after wounding for each of siCT or siRNA Hook2 (H1, H2, H1 + H2) transfected MCF7 cells in the same conditions as above. Cells were allowed to migrate for 15 hours (Bar 10 μm). Note in Hook2 depleted cells that the Golgi apparatus is compacted compared to control cells. Images shown are representative of siCT and siHook2 (H1 + H2) cells. The arrow shows the direction of cell migration. Schematic indicates analysis method to determine Golgi apparatus offset. (**d**) The percentage of cells with the Golgi apparatus in the forward facing 120 °C sector was measured in first row of cells adjacent to the scratch. For each experiment, 100 cells were scored. When the Golgi apparatus position could not be determined it was categorized as such (N.D.). Results shown are the mean of 3 independent experiments (*n* = 306 cells; *p = 0.0158; **p = 0.0085) for siCT and siHook2 (H1 + H2).

To analyze the effect of Hook2 on polarized migration, the position of the migration front was recorded every hour to calculate the velocity of the advancing leading edge. For each experiment Hook2 levels were measured in parallel by immunodetection on western blots to ensure the efficiency of the siRNAs. A slight delay in migration upon Hook2 depletion was observed in independent experiments without preventing an eventual closure (Fig. 1a,b). These results indicate that Hook2 may play a role, but it is not critical for migration or that it was not sufficiently depleted below a threshold level that would result in a more robust migration defect.

We next tested whether Hook2 affects the polarization of migrating MCF7 cells by monitoring the Golgi position as a marker to follow cell polarization. MCF7 cells were transfected with siRNA control (siCT) or siHook2 and monitored for changes in the positioning of the Golgi after 15 h in migrating MCF7 cells using TGN46 as a marker (Fig. 1c). The percentage of cells that were unable to correctly orient their Golgi apparatus (as defined in Fig. 1d) increased from 20% in control cells to above 50% in Hook2 depleted cells (Fig. 1d). These data indicate that Hook2 is necessary for the correct reorientation of the Golgi complex in polarized migrating MCF7 cells, a process that normally involves dynein^18^. In mammals, no direct binding between dynein and Hook2 has been described, but overexpression of a dominant negative form of Hook2 induced a phenotype similar to dynein loss of function in mouse cells indicating a possible common pathway for Hook2 and dynein^17^. To know if these results were MCF7 specific or general to other cell lines we performed the same experiments with MCF10A cells, which migrate more rapidly than MCF7 cells with wounds closed after 15 hours. A 70% depletion of Hook2 with two siRNA (H1 + H2) induced a significant delay in cell migration and a drastic miss-orientation of Golgi apparatus (Fig. S2). This data thus reinforces the important role of Hook2 in polarized cell migration.

Among the proteins that regulate polarized cell migration, PAR6 has been shown to regulate centrosome reorientation during polarized cell migration^4^. Overexpression of the N-terminal domain of PAR6α induced a disruption in Golgi orientation in migrating astrocytes^19^ similar to Hook2 depletion, suggesting that PAR6 and Hook2 may act in the same pathway to orient the MTOC and Golgi complex during polarized migration. To confirm this hypothesis we observed that in MCF7 migrating cells and over-expressing human Myc-PAR6α there is a co-localization between Hook2 and Myc-PAR6α at cell protrusions indicating a possible link between the migrating front and the centrosome (see supplementary Fig. S3). How PAR6α is able to control Golgi orientation in migrating cells is unclear, but it has been proposed that it may be mediated through its interaction with aPKC and microtubules^20^. Since Hook2 binds to microtubules through its N-terminal domain we thus investigated a potential functional interaction between Hook2 and PAR6α.

### Hook2 is essential for centrosome localization of PAR6α

**Figure 2:**
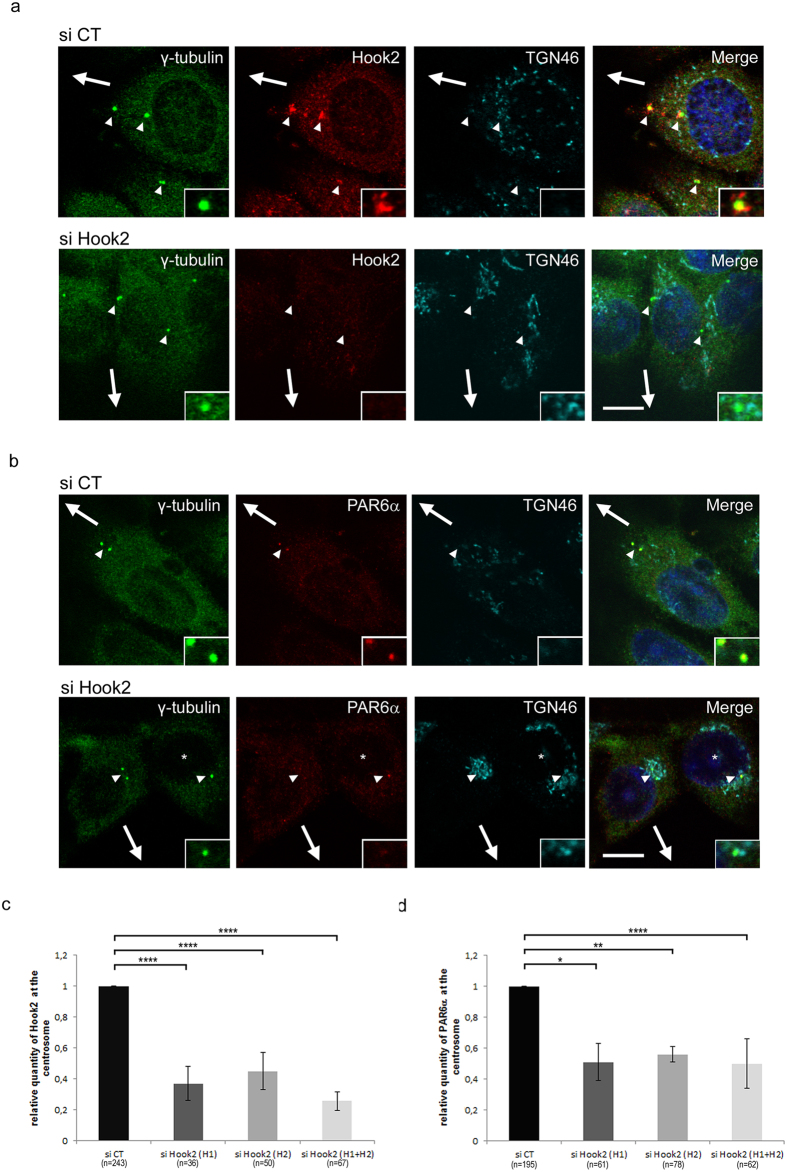
Hook2 is essential for PAR6α localization at the centrosome in migrating MCF7 cells. (**a**,**b**) MCF7 cells transiently transfected with siRNA control (siCT) or Hook2 (siHook2) for 3 days were co-stained with antibodies against γ-tubulin (to visualize the centrosome), TGN46 (to visualize the Golgi apparatus and more precisely its compaction due to Hook2 depletion) and Hook2 (in **a**) or PAR6α (in **b**) after methanol fixation. The arrowheads point to the centrosome and arrows indicate direction of migration. Bars = 10 μm and insert magnification, x5000. In the b lower panel, the asterisk indicates one cell non-transfected by siHook2 seen by the dispersed TGN46 labelling, note presence of PAR6α at the centrosome of this cell in comparison of its absence at the centrosome of adjacent transfected cell. (**c**,**d**) Quantification of immuno-localization of Hook2 (**c**) or PAR6α (**d**) at the centrosome in migrating transfected MCF7 cells (siCT and siHook2) in 3 independent experiments (****p < 0.0001; **p = 0.0087; *p = 0.0204).

Immunofluorescence staining and confocal microscopy analysis of Hook2 and PAR6α in migrating MCF7 or MCF10A (Fig. 2a,b; Fig. S4a,b) and resting MCF7 (Fig. S5a,b) cells showed that these two proteins localized to the centrosome, with some additional staining in the Golgi area for Hook2 as previously described in ARPE-19 cells^15^.

To address whether Hook2 plays a role in recruiting PAR6α to the centrosome during polarized cell migration, Hook2 was depleted from MCF7 or MCF10A cells in a wound healing assay and the centrosomal localization of PAR6α was quantified (Fig. 2d, Fig. S4d). First as expected from the Golgi complex orientation defect observed in Fig. 1b, centrosomes are no longer aligned with the migration direction upon Hook2 depletion (see for example Fig. 2a lower panel). Second, despite the fact that some Hook2 was still present at the centrosome after depletion (Fig. 2c, Fig. S4c) we found that PAR6α accumulation at the centrosome decreased to 53% or to 60% in, respectively, Hook2 (H1 + H2) KD MCF7 or MCF10A cells when compared to control experiments (Fig. 2d, Fig. S4d). Unfortunately, we could not perform co-localization experiments between Hook2 and PAR6α since both antibodies were generated in rabbit, however we were able to distinguish cells depleted of Hook2 by their characteristic Golgi condensation (TGN46 staining) as previously described^15^. The overall PAR6α protein levels in immunoblots performed on control and Hook2-depleted cells were indistinguishable, which indicates that the decreased signal found at the centrosome was compensated by a cytoplasmic contribution or that it represents a percentage too low to be quantified on immunoblots. Reciprocal experiments to eliminate PAR6α function were unsuccessful despite testing many different siRNAs. It is however likely that Hook2 localization at the centrosome might be independent of PAR6α, Hook2 has been shown to be anchored to the centrosome through interactions between its C-terminal domain and centrosomal proteins such as CENP-F and centriolin^16^,^21^.

### Hook2 interacts with PAR6α

**Figure 3:**
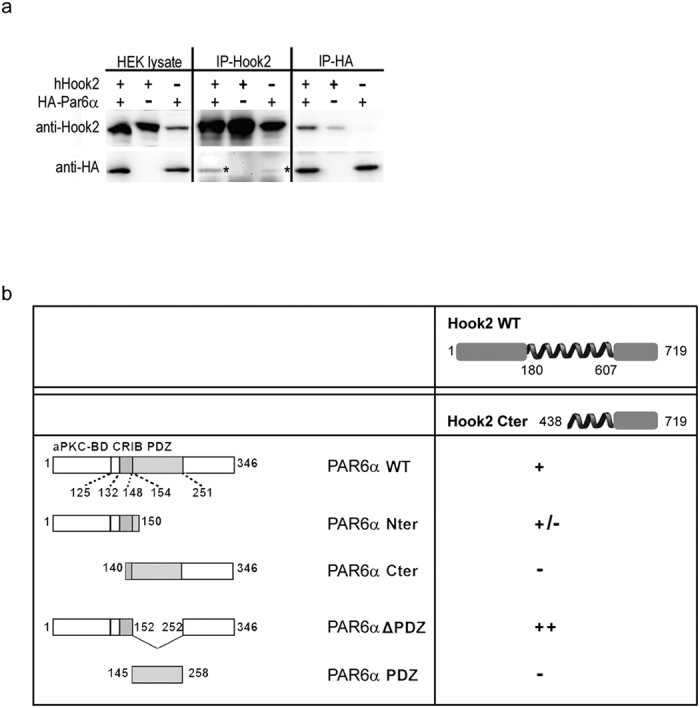
Hook2 interacts with PAR6α. (**a**) HEK cells transiently transfected with HA::PAR6α and/or human Hook2 plasmids were lysed (Lysate) after one day and their products were immunoprecipitated with mouse anti-HA antibodies (IP-HA) or rat anti-Hook2 (IP-Hk2) and processed for immunoblotting with rabbit anti-Hk2 and anti-HA antibodies. Asterisks indicate HA-PAR6α co-immunoprecipitated with overexpressed human Hook2 or endogenous human Hook2. (**b**) Hook2 Cter domain (amino acids 438 to 719) interacts with PAR6α Nter domain in two-hybrid assays. Hook2 Cter interacts (+) with PAR6α WT, Nter, and ΔPDZ but not (−) with PAR6α Cter and PAR6 PDZ motif.

To test whether PAR6α and Hook2 interact *in vivo*, we co-transfected human Hook2 and human HA-PAR6α in HEK cells and found these two proteins were associated in reciprocal co-immunoprecipitation experiments (Fig. 3a). To dissect this interaction on the molecular level we performed two-hybrid assays using the C-terminal portion of Hook2 (Hook2 Cter) and several PAR6α variants with different known functional domains (Fig. 3b). Hook2 Cter interacts with the full length PAR6α and with a stronger affinity to a PAR6α deleted of the PDZ domain. In addition a much weaker binding was observed when we used the PAR6α N-terminal domain (PAR6α Nter) and no binding at all was found with the PAR6α Cter suggesting that a change in conformation must occur for an efficient binding between Hook2 Cter and PAR6α Nter domains (Fig. S6). It is worth to note that this region of PAR6α encompasses the aPKC binding domain and the semi-CRIB domain (Fig. 3b).

### Hook2 acts as an adaptor to recruit the PAR complex

**Figure 4:**
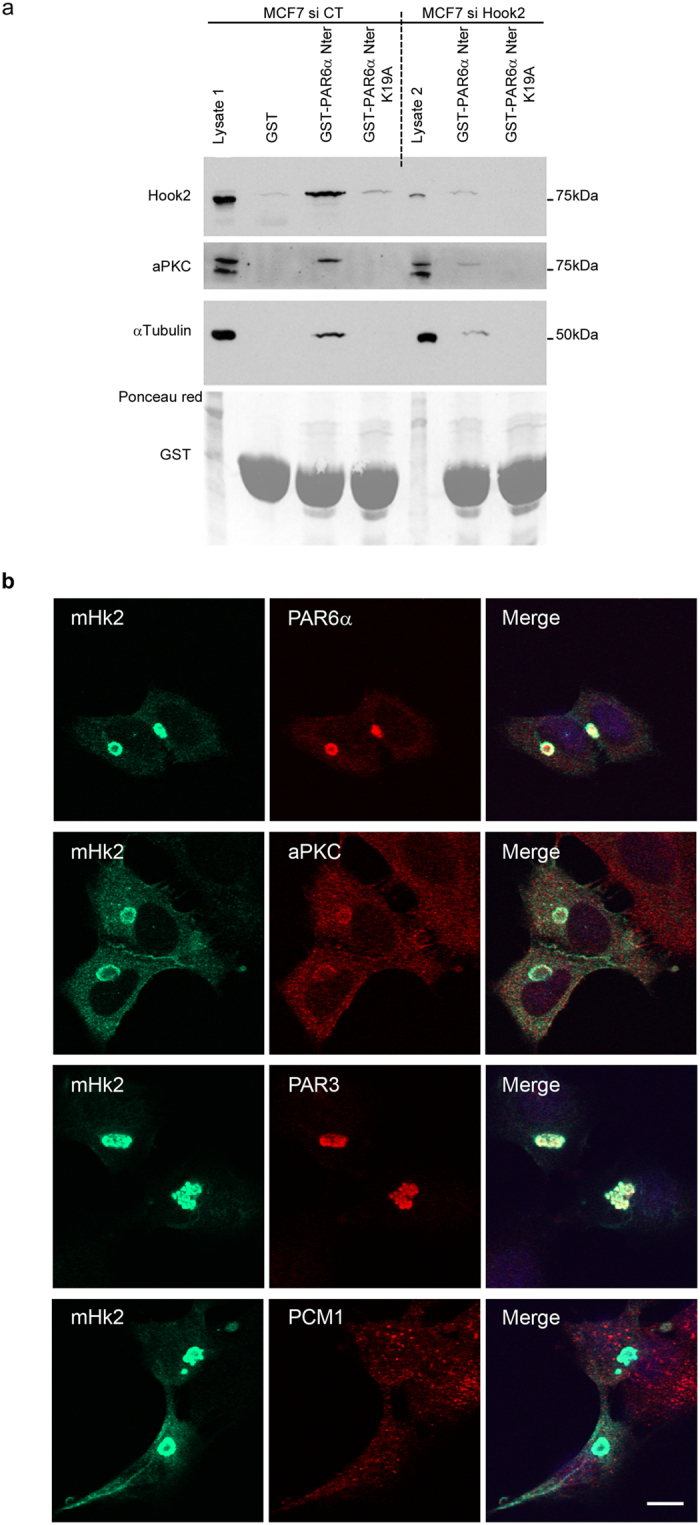
Hook2 acts as an adaptor for the PAR complex. (**a**) Hook2 mediates aPKC binding to PAR6α. siCT or siHook2 RNA transiently transfected MCF7 cell (3 days) lysates were used to perform GST-pull down assays with the GST-PAR6α Nter (wild type or K19A) fusion proteins (called GST-PAR6α Nter and GST-PAR6α Nter K19A) or GST alone. The Ponceau red-stained blots (lower panel) show the relative quantity of GST constructs used in each lane. (**b**) Exogenous mouse Hook2 recruits the PAR6α/PAR3/aPKC complex. MCF7 cells were fixed by ethanol/acetic acid after transient overexpression of mouse Hook2 (mHk2) for 16 hours. mHk2 is visualized with Hook2 rat antibodies (in green) and PAR6α or aPKC or PAR3 or PCM1 by rabbit polyclonal antibodies (in red). Note that PCM1 does not accumulate at the aggresomes in contrast to PAR6α, PAR3 and aPKC. Bars = 10 μm.

To better define the interaction between PAR6α and Hook2, a GST fusion protein containing the aPKC-binding domain of PAR6α (GST-PAR6α Nter) was generated and incubated with human epithelial cell lysates (Fig. 4a). Hook2 co-precipitated with GST-PAR6α Nter together with aPKC, a known partner of PAR6^22^,^23^ while it did not bind to GST alone. Our observations indicated that Hook2 and aPKC might thus bind to the same domain of PAR6α. Therefore we used a mutation in PAR6α Nter (K19A) that abrogates aPKC binding^24^ to test whether this affected the ability of PAR6α to bind to Hook2. Indeed, when we performed the same GST-PAR6α Nter K19A pull-down experiments, Hook2 binding was abrogated indicating that both Hook2 and aPKC require an intact aPKC binding domain in PAR6α further reinforcing our finding of a specific binding between the two proteins (Fig. 4a). We next questioned whether aPKC might require Hook2 to associate with this domain of PAR6α. Towards this aim we depleted Hook2 from cells and repeated our GST-PAR6α Nter pull-down assays. The levels of GST-PAR6-bound aPKC were much lower in the precipitates suggesting that Hook2 plays a role in bridging aPKC to PAR6α or facilitating their binding (Fig. 4a).

**Figure 5:**
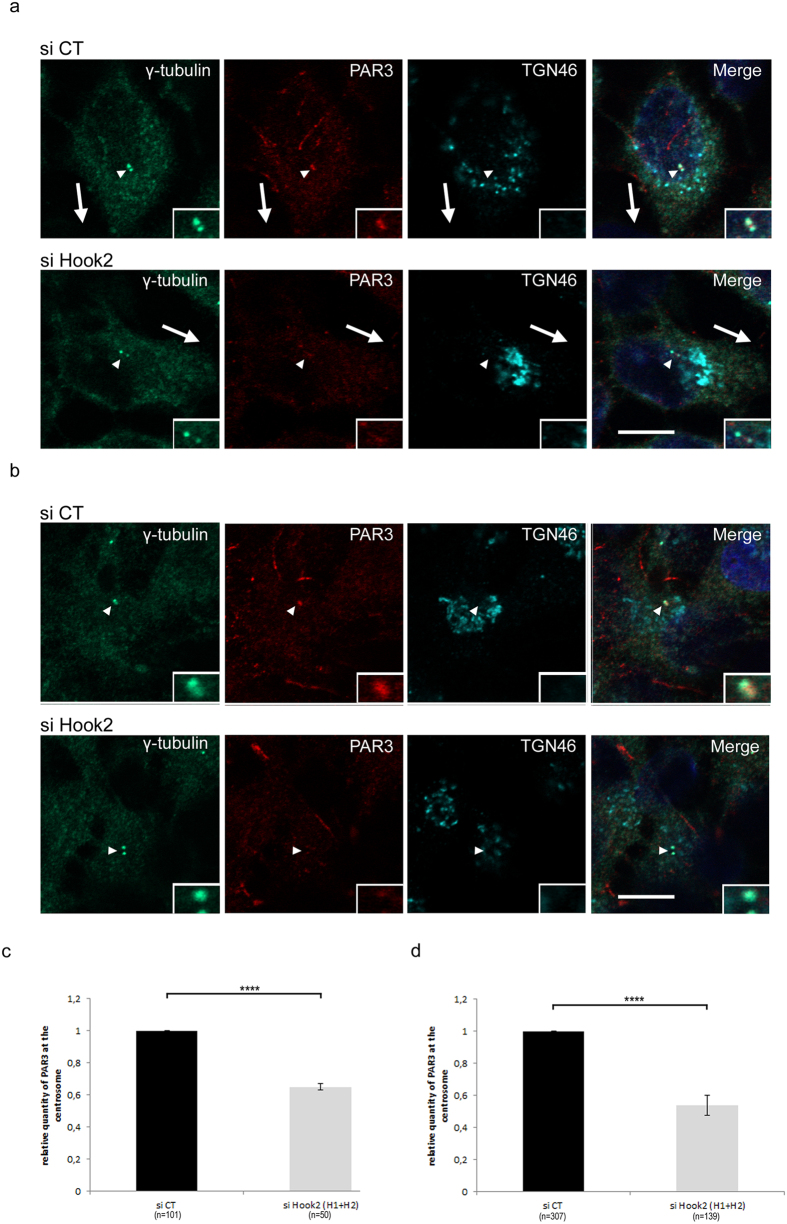
Hook2 is essential for PAR3 localization at the centrosome in MCF7 cells. (**a**,**b**) MCF7 cells transiently transfected with siRNA control (siCT) or Hook2 (siHook2) for 3 days were co-stained with antibodies against γ-tubulin, TGN46 and PAR3 after methanol fixation in migrating (**a**) or resting (**b**) MCF7 cells. The arrowheads point to the centrosome (in **a**,**b**) and arrows indicate direction of migration (in **a**). Bars = 10 μm and insert magnification, x5000. (**c**,**d**) Quantification of immuno-localization of PAR3 at the centrosome in migrating (**c**) or resting (**d**) transfected MCF7 cells (siCT and siHook2) in 3 independent experiments (****p < 0.0001).

**Figure 6:**
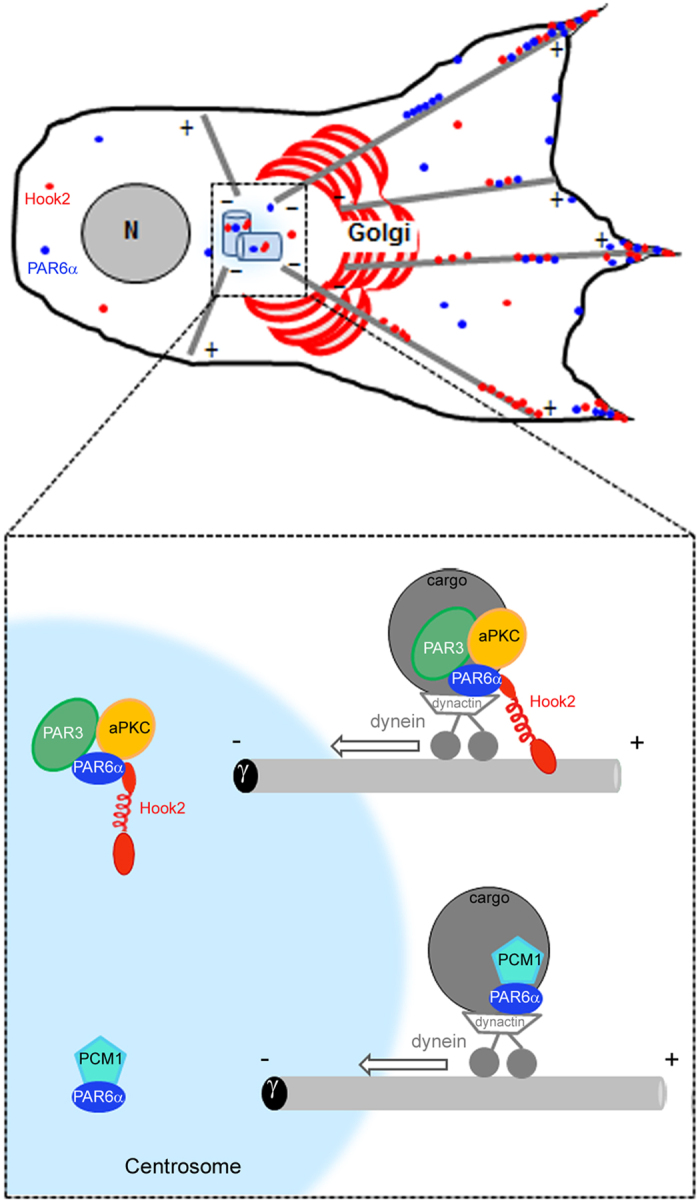
Model for PAR6α recruitment at the centrosome. In the migrating cell, Hook2 and PAR6α are represented by red and dark blue color, respectively. The upper part of magnification of centrosomal region illustrates participation of Hook2 in transport of the PAR6 complex to the centrosome via dynein/dynactin complex and in its stabilization. In contrast, the lower part illustrates Hook2-independent targeting of PCM1-PAR6α as suggested by the work of Kodani *et al*.^6^.

Our data so far support the existence of an important role of Hook2 for centrosomal PAR6α location, but also a more surprising role for the interaction between PAR6α and aPKC. To confirm this role of Hook2 in building a PAR6α-aPKC complex we over-expressed a mouse Hook2 (mHook2) in MCF7 cells which was previously found to induce aggresome formation in the peri-centrosomal area^17^ where Hook2 is found to accumulate (Fig. 4b). Strikingly PAR6α, aPKC and PAR3 strongly co-localized with m Hook2 at the aggresome, but we did not observe PCM1, despite its ability to bind to Hook2^15^. This specific recruitment of PAR6α, aPKC and PAR3 by over-expressed mHook2 suggests that Hook2 might act as an adaptor to localize the PAR complex. Indeed when we monitored the accumulation of PAR3 to the centrosome in Hook2 depleted MCF7 cells either migrating (Fig. 5a) or resting (Fig. 5b) we could detect a significant decrease of PAR3 association to the centrosome (Fig. 5c,d). That a cellular association between Hook2 and the PAR3/6 complex exists is reinforced by the fact that we observed Hook2 phosphorylation *in vitro* by aPKC and it has been reported that two Hook2 peptides are indeed phosphorylated *in vivo* in the coiled-coiled domain^25^. This Hook2 role seems to be independent of PCM1 because both PCM1 levels and localization are unaffected by Hook2 over-expression or depletion in MCF7 cells (Fig. S7). This is surprising since an association between PAR6α and PCM1 at the centrosome and the peri-centriolar material was identified in human Hela cells^6^. We also uncovered that in human ARPE-19 cells, Hook2 forms a complex with PCM1 and its depletion induced a degradation of PCM1^15^. Together this data favors a model in which Hook2 acts through PAR6α to recruit the PAR complex to the centrosome in parallel to an alternative recruitment of PCM1/p150^Glued^ by PAR6α (see model in Fig. 6 and Kodani *et al*.^6^). Further work will help to clarify if Hook2 forms cell-type specific complexes with PCM1 and PAR6α and the respective contribution of the two mechanisms to recruit PAR6α to the centrosome. In addition, PAR6γ has been shown to recruit PAR6α to the centrosome^26^ and further work will be necessary to investigate the relationship between Hook2 and PAR6γ.

In conclusion, the interactions between Hook2 and PAR6α/PAR3/aPKC (this study) and centriolin or CENP-F in mammalian epithelial cells^16^ and the recruitment of dynein by the C. elegans Hook homologue, ZYG-12, favor a model wherein Hook2 acts as a link between the PAR complex and the MTOC^27^ (see Fig. 6). The precise dissection of the spatio-temporal regulation that control the dynamics of the PAR6α/PAR3/aPKC interactions with Hook2 will be a major focus of investigation to better understand how these complexes contribute to the dynamic process of epithelial migration in the future.

## Methods

### Cell culture and transfections

MCF7 (human breast carcinoma cell line) and HEK cells were grown, respectively, in RPMI or DMEM-glutamax supplemented with 10% fetal bovine serum. The human breast non-tumorigenic epithelial MCF10A cell line was maintained in complete medium, DMEM: F12 (Dulbecco’s Modified Eagle Medium: F12) supplemented with 5% Horse Serum, 1% non-essential amino acids, 10 ng/ml EGF, 10 μg/ml Gentamycin (all from Invitrogen), 10 μg/ml Insulin, 1 μg/ml Hydrocortisone, 100 ng/ml Cholera Toxin (all from Sigma Aldrich, St Louis, MO, USA), and cultured at 37 °C with 5% CO2. MCF7 cells were transfected with 100 pmol of siRNA or 2 μg of plasmid using nucleofector kit V and program P-020 of AMAXA Biosystem according to the manufacturer’s instructions (AMAXA). MCF10A were transfected with 100 pmol of siRNA using nucleofector kit T and program T-020 of AMAXA Biosystem. Control siRNA against a sequence of Luciferase (5′cguacgcggaauacuucga3′), Hook2 siRNA H1 (ID#20301: 5′ggagacucugauuuuauau3′) and H2 (ID#20392: 5′ggaccacauccagagaauc3′) were from Ambion/Applied Biosystems (Courtaboeuf France). siRNA H1 and H2 were transfected together or separately, Hook2 depletions were quantified by western blotting and the same phenotypes for these two siRNA were obtained. Full-length cDNA encoding mouse Hook2 was described previously^9^. Over-expression of mouse Hook2 in MCF7 cells was obtained after reverse transfection with Fugene HD reagent (Roche) using the manufacturer’s instructions. The cDNA encoding full length human Par6α was obtained by RT-PCR on a human breast library and subsequently cloned into pcDNA3.1/Myc-His vector (invitrogen) with EcoRI and XbaI cloning sites to obtain a Myc-Par6α.

Full-length cDNA clone (BC012443) of human Hook2 was from I.M.A.G.E. consortium. HEK cells were transfected with human Hook2 and HA::PAR6α cDNA (given by Dr. IG Macara University of Virginia School of Medicine, Charlottesville, VA, USA) together or alone using nucleofector kit V and program A-023 of AMAXA Biosystem according to the manufacturer’s instructions (AMAXA).

### Protein interactions and two-hybrid assays

For co-immunoprecipitations, HEK cells transiently expressing human Hook2 and HA::PAR6α (during one day) were lyzed and processed as previously described^15^,^28^. For GST pull downs, MCF7 cells transfected with CT or Hook2 siRNA were lysed after 3 days, revealed and quantified as previously described^29^. GST-PAR6α Nter (1–133 amino acids) was generated by PCR from PAR6α template using high fidelity Taq polymerase (Invitrogen) and was inserted as a BamHI/EcoRI fragment into pGEX4T3. For the yeast two-hybrid assay, we used L40 yeast and a LexA system with human Hook2 Cter (amino acids from 438 to 719, a BamHI/BamHI fragment) in pBTM116 as bait and human PAR6α WT, PAR6α Nter, PAR6α PDZ, PAR6α ΔPDZ, PAR6 Cter inserted in the pACT2 vector (BD Biosciences) as target of interaction have already been described^30^. Then, yeast transformed (growing in the absence of tryptophan and leucine) clones were harvested after selection in the absence of tryptophan, leucine, and histidine and in the presence of 10 mM and 5 mM of 3-aminotriazole. We used a second system of selection on X-Gal medium.

### Immunofluorescence staining, confocal microscopy and quantification

Cells were grown on glass coverslips and processed as described previously^31^. For immunofluorescence experiments, siRNA transfected MCF7 or MCF10A cells were seeded on glass coverslips, maintained for 3 days in culture. Cells were fixed in methanol for 4 minutes at −20 °C (for centrosomal labeling) or ethanol 95%, acetic acid 5% (for aggresome labeling) for 6 minutes at 4 °C or 3% paraformaldehyde/PBS (for Golgi labeling) for 25 minutes then permeabilized 10 minutes in 1% Triton X-100/PBS (depending on the antibody used). Rabbit polyclonal antibody against Hook1, 2 and 3 were described previously^9^ and rat polyclonal antibody against Hook 2 were custom made using the same peptide as in ref. 9. Rabbit polyclonal anti-PAR6α was a gift from Dr Lammers (Tübingen, Germany). Antibodies used are rabbit polyclonal antibody against aPKC (Santa Cruz Biotechnology), against PAR3 (Upstate), against PCM1 (Bethyl laboratories), a sheep polyclonal antibody against TGN46 (AbD Serotec), mouse monoclonal antibody against α-tubulin (Sigma clone B-5-1-2), against γ-tubulin (Sigma clone GTU-88), against HA tag (Abcam, clone 16B12). Images were taken using a Zeiss 510 Meta confocal microscope (Zeiss) and were analyzed as following. Centrosomes of cells were detected using γ-tubulin fluorescent signals in the immunostaining images. Using custom written Matlab codes based on the regionprops function, all the centrosomes within an image were automatically segmented and localized. Hook2, PAR6α and PAR3 fluorescent intensities were then automatically measured in the precise localization of the segmented centrosome.

### Wound repair assay

MCF7 or MCF10A cells were grown to confluence three days after transfection. The medium was changed 16 hours before scraping the cell monolayer with a P200 micropipette tip. The scratches were normally closed in about 48 hours. For cell migration speed experiments, immediately after the scratch, cells were placed in the 37 °C, 5% CO2 incubator box of a Zeiss Colibri microscope (Zeiss, Le Pecq, France). We used a multidimensional time-lapse acquisition with Axio vision software at low magnification (x10) and in phase contrast. Each acquisition was of 100 milliseconds and was separated from the next by 1 hour during 48 hours. The distance of migration at each time point, at each pixel of the migrating front, for each siRNA was analyzed using a custom written MatLab codes based on the bwdist function that computes the Euclidean distance between the nearest nonzero-labelled pixel.

### Statistics

Results are expressed as means and standard deviations. Statistical analysis were performed with GraphPad Prism 6 and based on an unpaired T-test analysis between the CT cells and Hook2 depleted cells.

## Additional Information

**How to cite this article**: Pallesi-Pocachard, E. *et al*. Hook2, a microtubule-binding protein, interacts with PAR6α and controls centrosome orientation during polarized cell migration. *Sci. Rep.* **6**, 33259; doi: 10.1038/srep33259 (2016).

## Supplementary Material

Supplementary Information
