# Supplementary material for: Hook2, a microtubule-binding protein, interacts with Par6α and controls centrosome orientation during polarized cell migration
Source: Sci Rep. 2016 Sep 14;6:33259. doi: 10.1038/srep33259 (PMC5021942; doi:10.1038/srep33259)
Supplement: Supplementary Information [file srep33259-s1.doc]

**Hook2, a microtubule-binding protein, interacts with Par6α and controls centrosome orientation during polarized cell migration.**

Emilie **Pallesi-Pocachard**^1,2,+^, Elsa **Bazellieres**^1,+^, Annelise **Viallat-Lieutaud**^1^, Marie-Hélène **Delgrossi**^1^, Magali **Barthelemy-Requin**^1^, André **Le Bivic**^1*^ and Dominique **Massey-Harroche**^1*^

1) Aix-Marseille Univ, CNRS, UMR 7288, Developmental Biology Institute of Marseille (IBDM), case 907. 13288 Marseille, cedex 09. France.

2) present address: Aix-Marseille Univ, Inserm UMR 901 (INMED), Campus de Luminy, 13288 Marseille Cedex 09, France.

^+^: Co-first authors

*: Co-senior authors and equal contribution

**Supplementary Figure 1:**


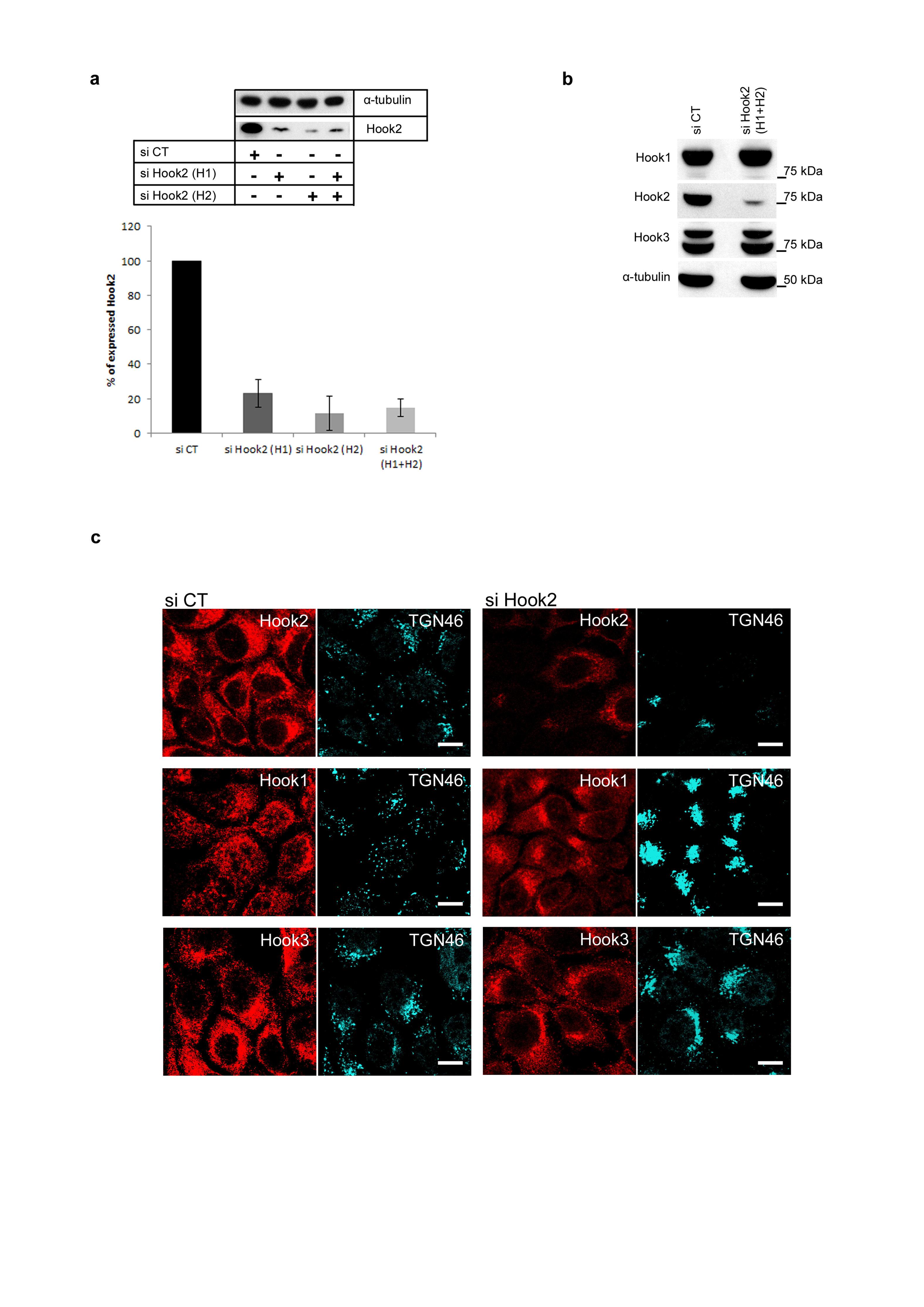


Figure S1: Two small interferent RNA reduce Hook2 expression

a- Two siRNA (H1 and H2) against Hook2 or a siRNA control (siCT) were transiently transfected in MCF7 cells. After 5 days, Hook2 was quantified using immuno-detection on Western blots with antibodies against Hook2, α-tubulin was used as a loading control. Relative levels of Hook2 protein in treated cells (siHook2: H1, H2 and H1+H2) are indicated as a percentage of control levels (mean ± s.d., *n*=5).

b, c- Depletion of Hook2 does not modify the expression levels of Hook1 or 3. MCF7 cells were transiently transfected with siHook2 (H1+H2) for 3 days and immuno-detected after Western blotting with specific antibodies against Hook1, 2 or 3. α-tubulin antibodies were used to monitor gel loads (b). Immunofluorescence labeling of Hook2 or Hook1 or Hook3 and TGN46 were performed with these cells (c). Note the characteristic compaction of the Golgi when Hook2 is depleted. Bars = 10µm.

**Supplementary Figure 2:**

**
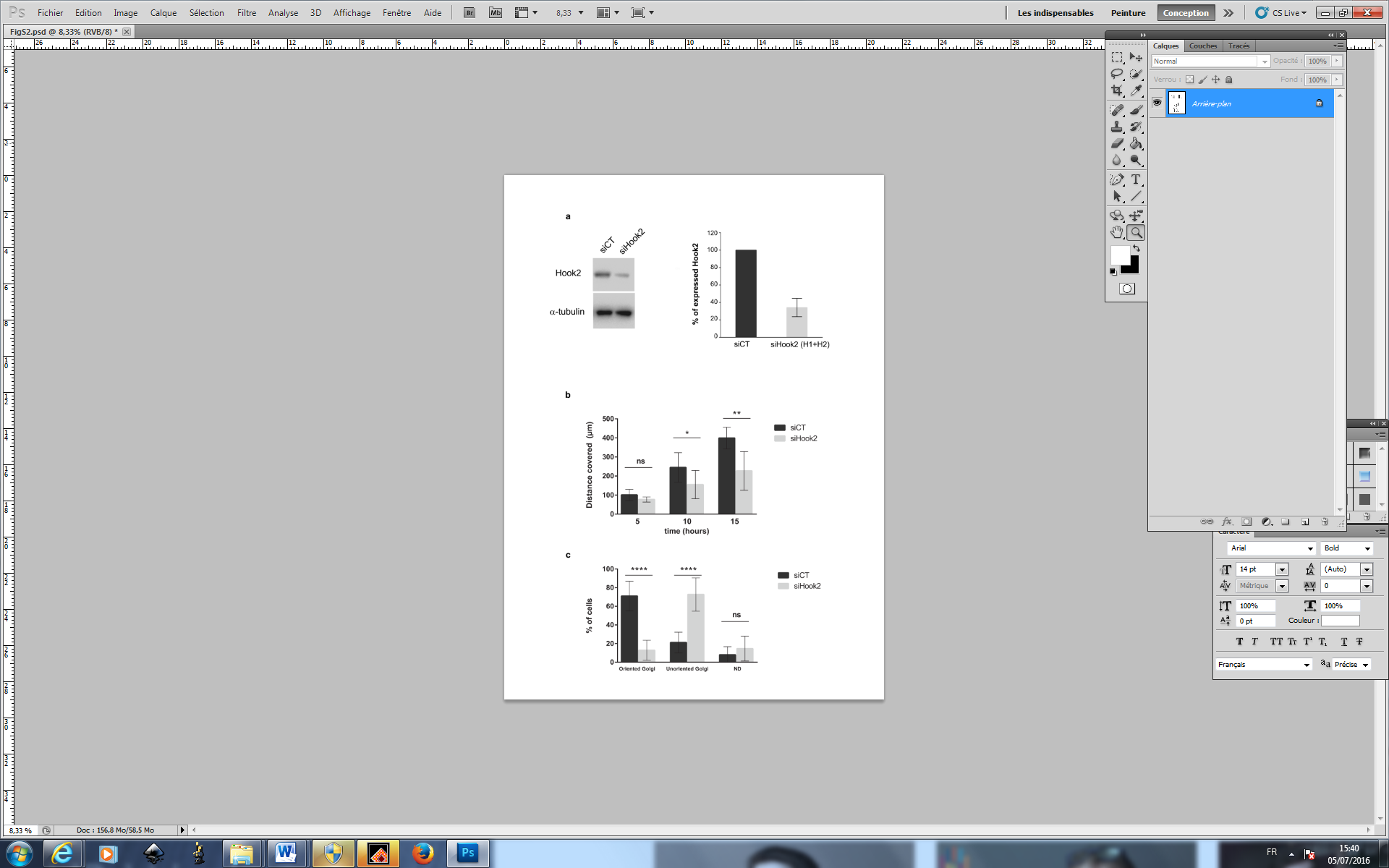
**

Figure S2: Hook2 controls Golgi orientation in MCF10A during polarized cell migration.

MCF10A cells were transiently transfected with siCT or siHook2 (H1+H2). a- After 5 days, Hook2 was quantified using immuno-detection on Western blots with antibodies against Hook2, α-tubulin was used as a loading control. Relative levels of Hook2 protein in treated cells (siHook2: H1+H2) are indicated as a percentage of control levels (mean ± s.d.; *n*=5). b- The estimation of cell-migration capacity was obtained by measurement of the distance covered 5, 10 and 15 hours after a scratch (*n*=3; *: p = 0.0293; **: p = 0.0073). c- Golgi reorientation was analyzed after wounding for siCT or siRNA Hook2 (H1+H2) transfected MCF10A cells in the same conditions as above. Cells were allowed to migrate for 10 hours (% of cells ± s.d.; *n*=5; ****: p<0.0001).

**Supplementary Figure 3:**


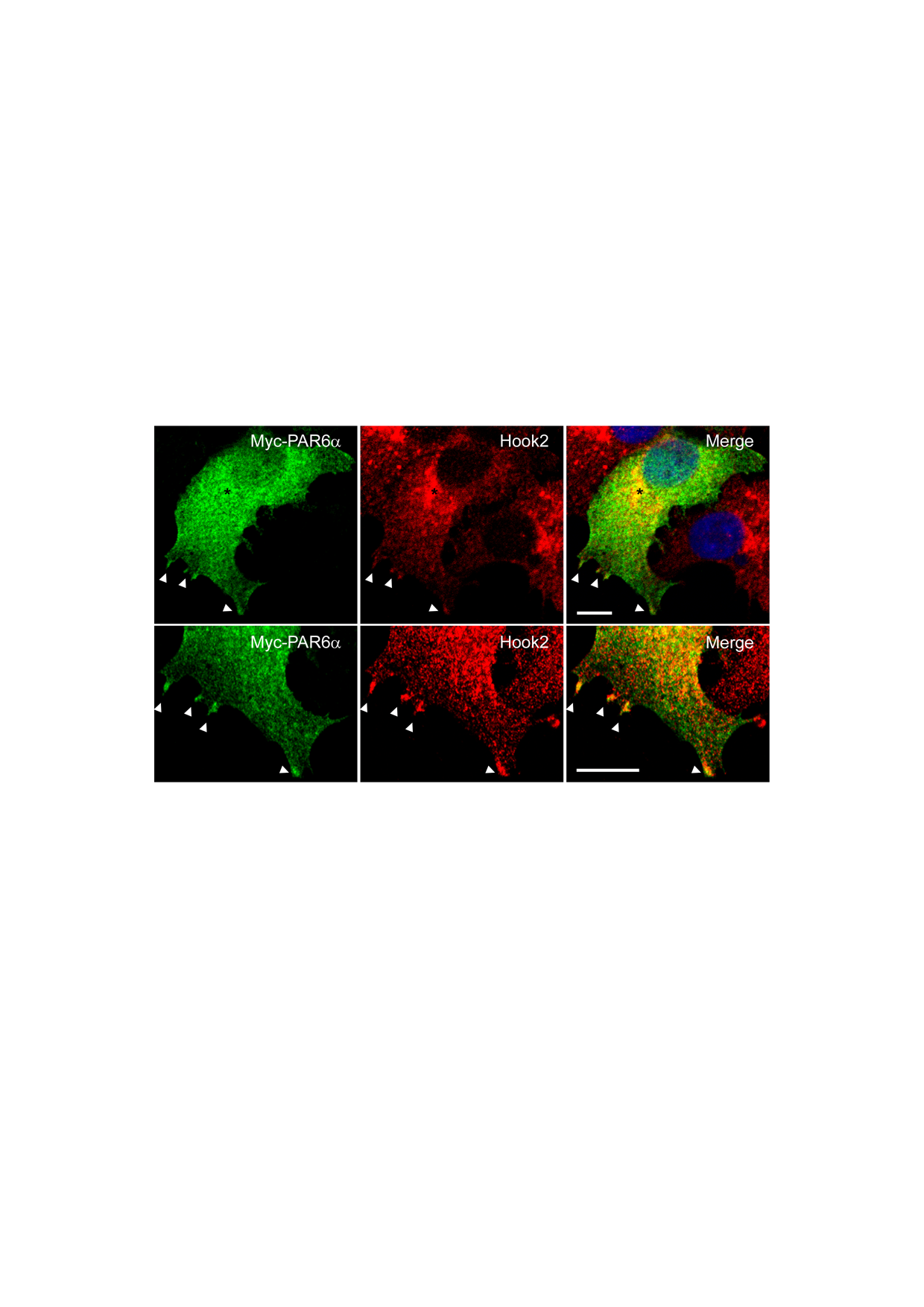


Figure S3: Hook2 and Myc-PAR6α co-localize at filopodia in migrating cells. MCF7 are transfected by Myc-PAR6α, after one day cells are fixed with paraformaldehyde and co-stained with antibodies against Hook2 and Myc. Asteriks indicate Golgi apparatus area. The arrowheads point to filopodia. Bars = 10µm.

**Supplementary Figure 4:**


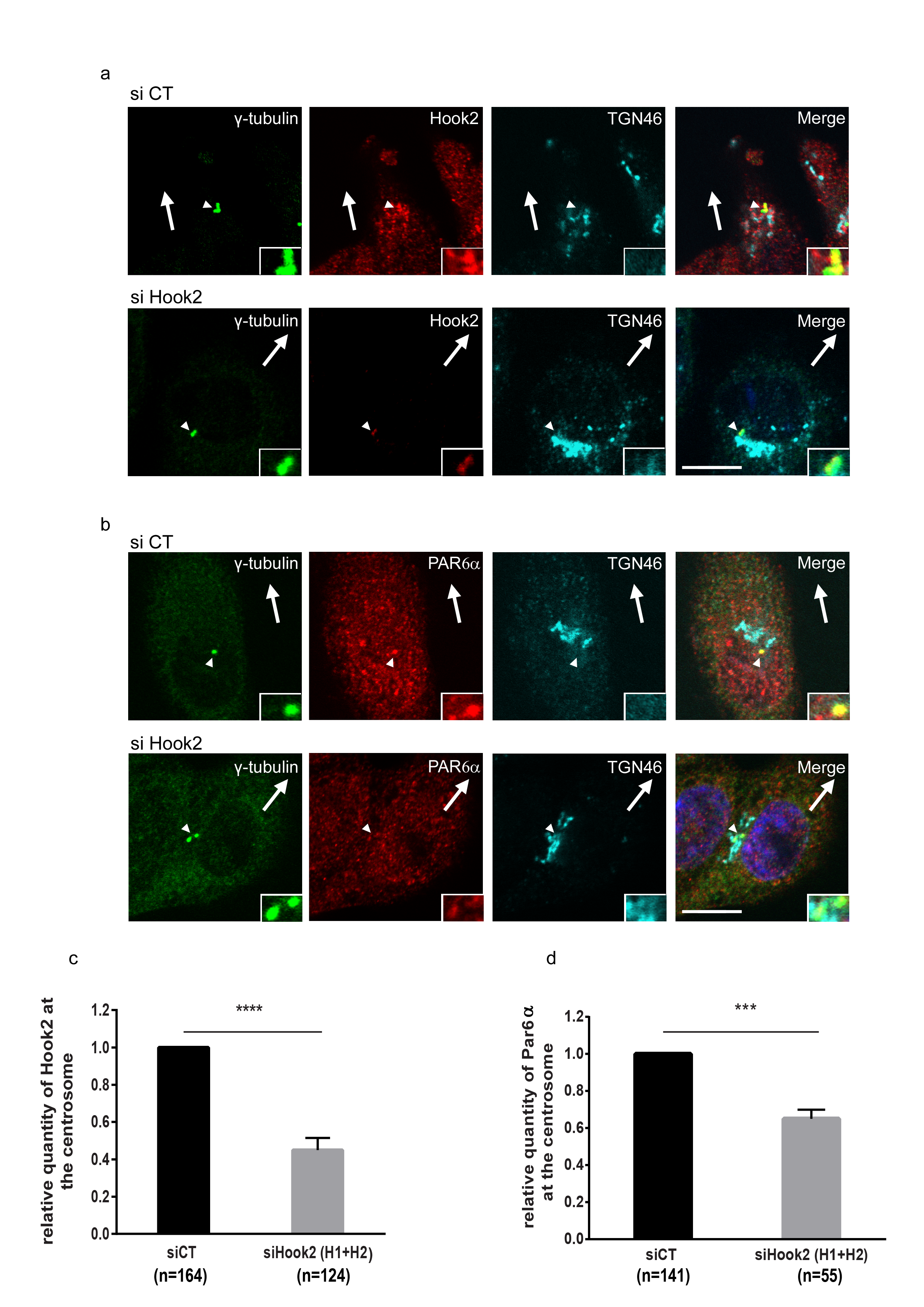


Figure S4: Hook2 is essential for PAR6α localization at the centrosome in migrating MCF10A cells

a, b- MCF10A cells transiently transfected with siRNA control (siCT) or Hook2 (siHook2: H1+H2) for 3 days were co-stained with antibodies against γ-tubulin (to visualize the centrosome), TGN46 (to visualize the Golgi apparatus and more precisely its compaction due to Hook2 depletion) and Hook2 (in a) or PAR6α (in b) after methanol fixation. The arrowheads point to the centrosome and arrows indicate the direction of migration. Bars = 10µm and insert magnification, x5000. c, d- Quantification of immuno-localization of Hook2 (c) or PAR6α (d) at the centrosome in migrating transfected MCF10A cells (siCT and siHook2) in 3 independent experiments (****: p<0,0001; ***: p=0,0003).

**Supplementary Figure 5:**


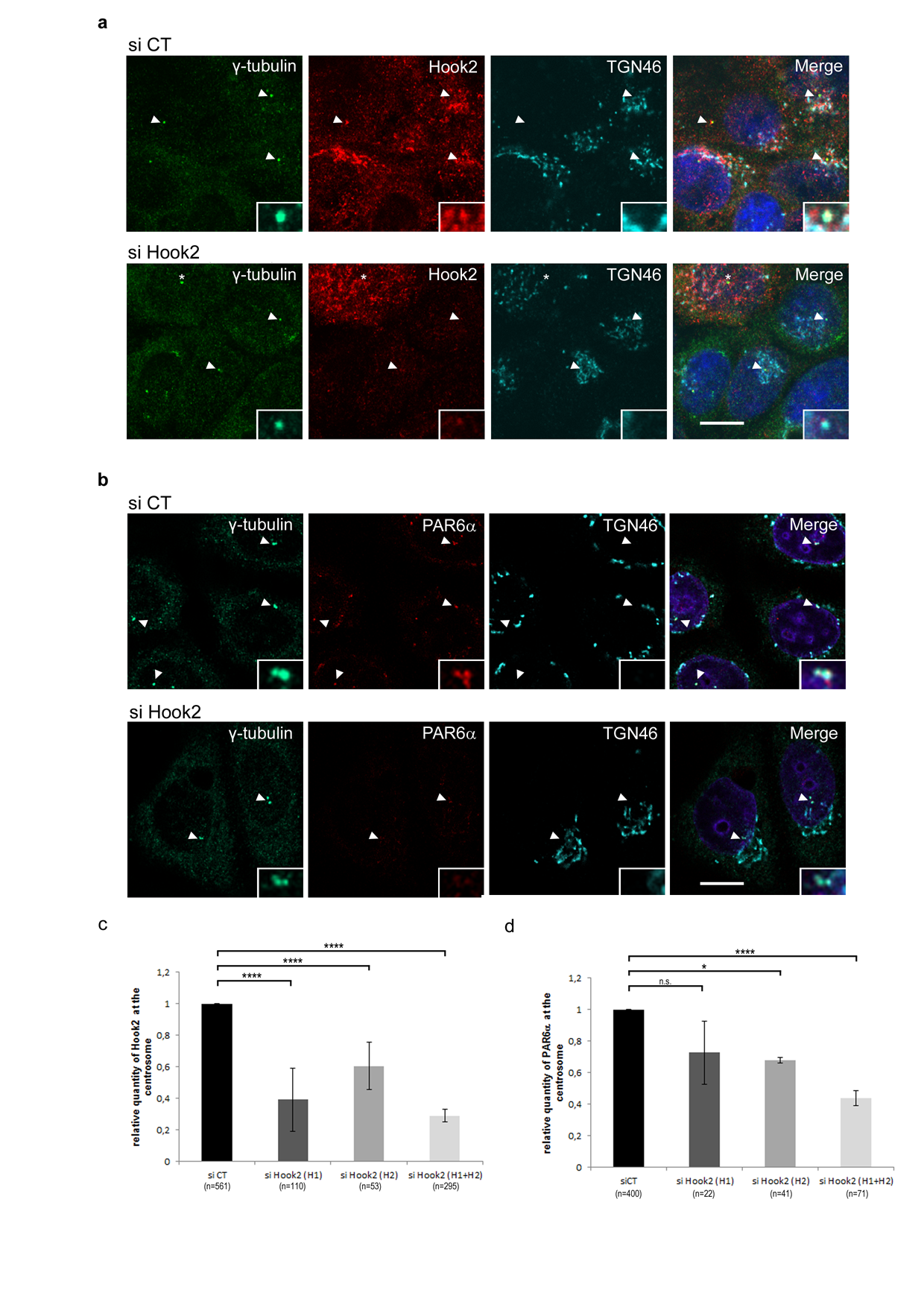
 Figure S5: Hook2 is essential for PAR6α localization at the centrosome in resting MCF7 cells. a, b- MCF7 cells transiently transfected with siRNA control (siCT) or Hook2 (siHook2) for 3 days were co-stained with antibodies against γ-tubulin, TGN46 and Hook2 (in a) or PAR6α (in b) after methanol fixation. The arrowheads point to the centrosome. Bars = 10µm and insert magnification, x5000. c, d- Quantification of immuno-localization of Hook2 (c) or PAR6α (d) at the centrosome in migrating transfected MCF7 cells (siCT and siHook2) in 3 independent experiments (****: p<0,0001; *: p=0,0353).

**Supplementary Figure 6:**


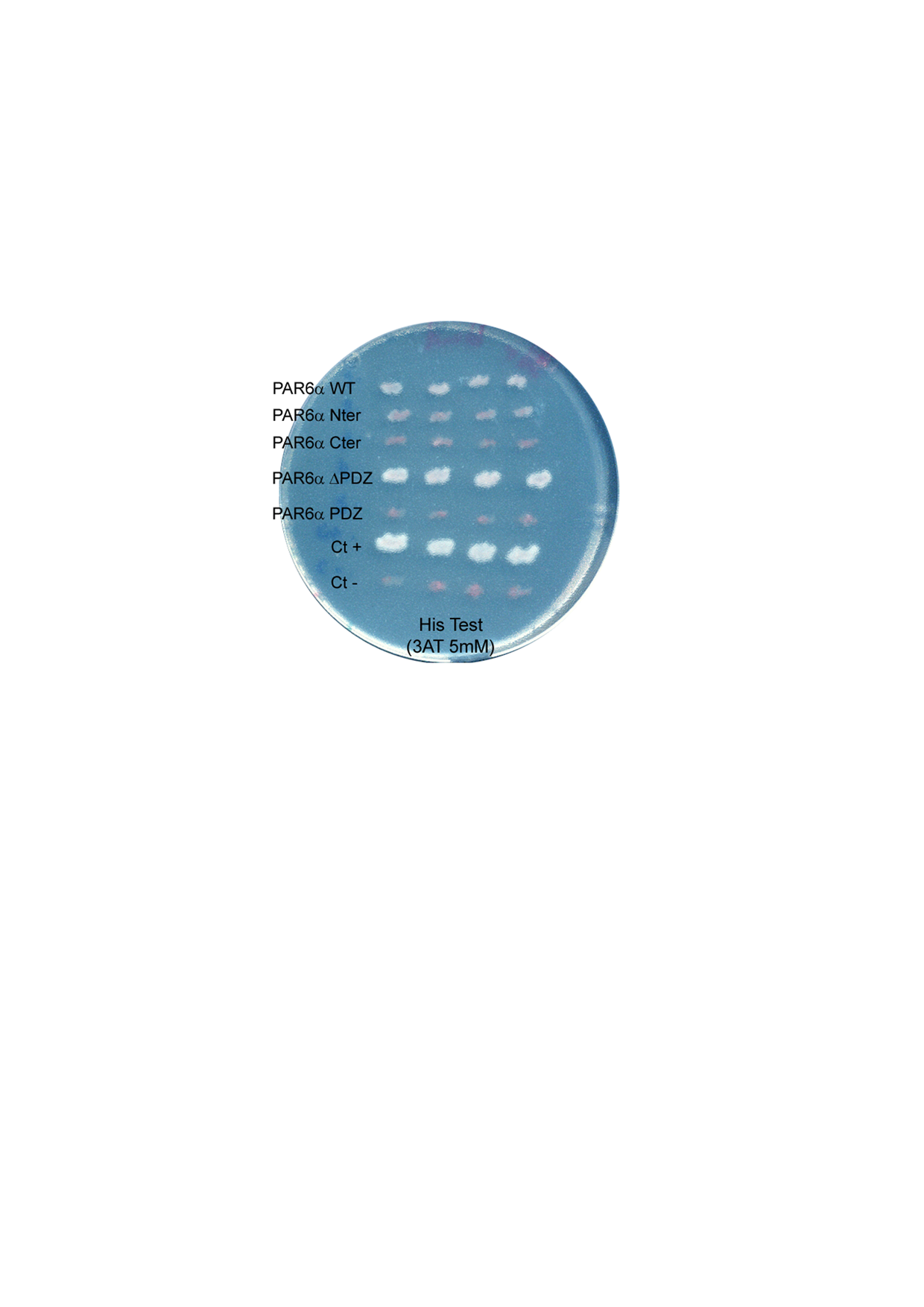


Figure S6: Hook2 interacts with the N-terminal domain of PAR6α through its carboxyl-terminal region. Y2H analysis of the interaction between carboxyl-terminal region of Hook2 and the whole PAR6α (WT), or N-terminal domain of PAR6α with or without PDZ motif (Nter or ΔPDZ, respectively), or its C-terminal (Cter) domains or only the PDZ motif of PAR6. Interactions are evidenced by the capacity of co-transformed L40 yeast to grow on plates depleted of histidine supplemented with 5mM 3-AT. Positive control (Ct+) is pBTM116-Her2 + pACT2-Erbin and negative control (Ct-) is pBTM116-Her2 + empty pACT2 vector used previously in Borg et al., Nat. Cell Biol., 2000, 2:407-414.

**Supplementary Figure 7:**


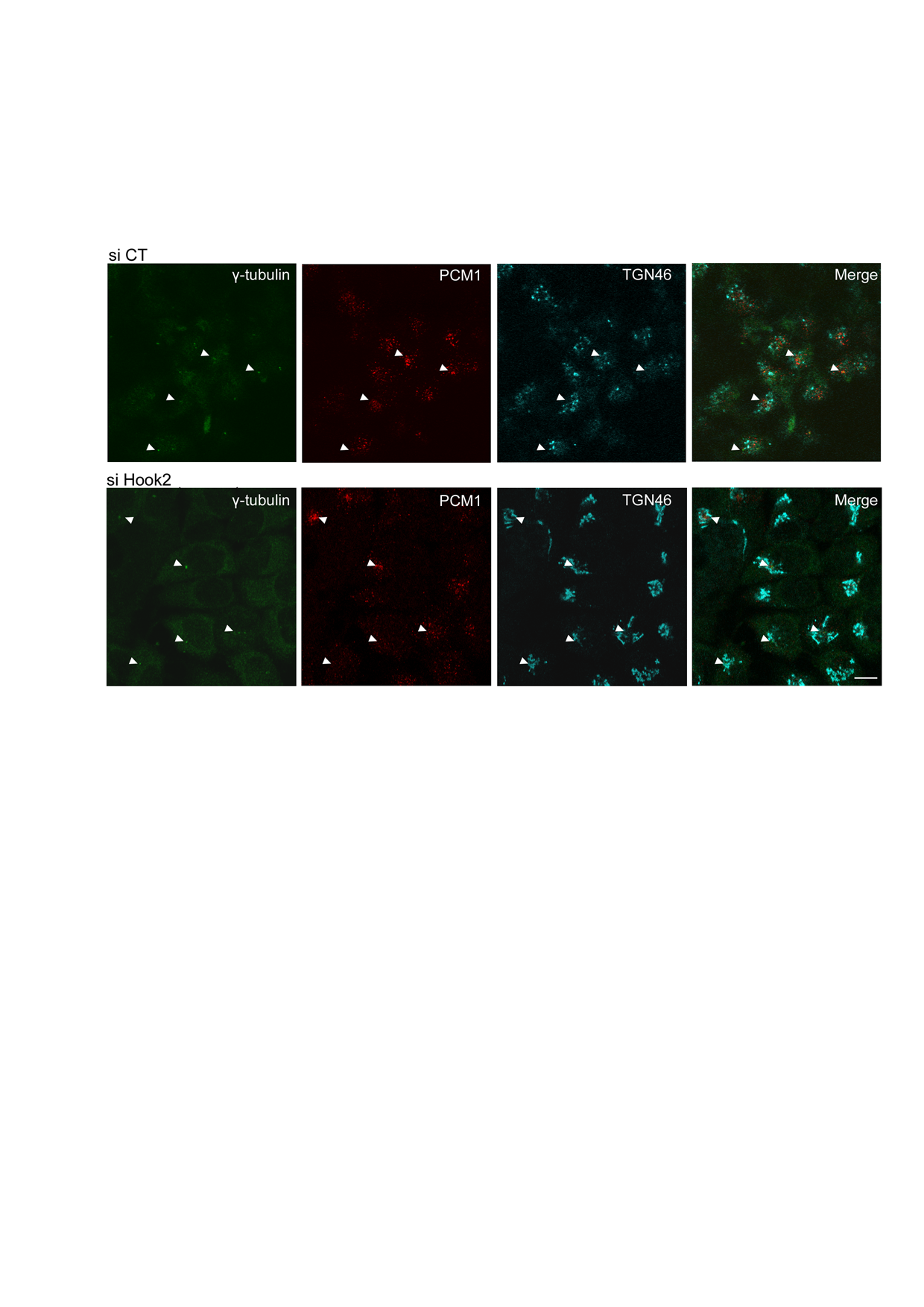


Figure S7: Hook2 depletion does not prevent PCM1 peri-centriolar localization. MCF7 cells transiently transfected with control (siCT) or Hook2 (siHook2) siRNA were co-stained with antibodies against γ-tubulin, PCM1, and TGN46. The arrowheads point to the centrosome. Bars = 10µm
